# Occurrence and Antimicrobial Resistance Profiles of *Escherichia coli* Isolated from Commercial Poultry Farm in West Kazakhstan

**DOI:** 10.3390/biology15110877

**Published:** 2026-06-02

**Authors:** Laura Dushayeva, Kenzhebek Murzabayev, Adilbay Karagulov, Kodjovi D. Mlaga, Rashid Karmaliyev, Askar Nametov, Bekzhassar Sidikhov, Aiman Ichshanova, Rasul Sidikhov, Gulzhan Demeugaliyeva, Miras Gabbassov

**Affiliations:** 1 Scientific Research and Production Laboratory of Veterinary and Biological Safety (BSL-2) Laboratory, Faculty Veterianry and Agrotechnology, Non-Profit Joint-Stock Company “Zhangir Khan West Kazakhstan Agrarian-Technical University”, Zhangir Khan Street 51, Uralsk 090009, Kazakhstansidihovbm@mail.ru (B.S.);; 2 The Microbiome and Mucosal Defence Research Unit, Institut de Recherche Clinique de Montréal, Montreal, QC H2W 1R7, Canada; 3 Research Institute for Biological Safety Problems LLP, «QazBioPharm» National Holding, 15 B. Momyshuly Street, Gvardeiskiy Village, Kordai District, Zhambyl Region 080409, Kazakhstan; sidihovrb@mail.ru

**Keywords:** *Escherichia coli*, antimicrobial resistance, resistance genes, mobile genetic elements, One Health

## Abstract

The widespread use of antimicrobials in poultry production exerts selective pressure for the emergence of resistant bacteria, yet molecular data on resistance determinants in *Escherichia coli* from commercial farms in Central Asia remain scarce. This study aimed to characterize antimicrobial resistance genes and mobile genetic elements in *E. coli* isolates from a layer poultry farm in the West Kazakhstan region. A total of 100 samples (cloacal swabs, fresh feces, internal organs, and wild pigeons) were collected. *E. coli* was isolated using selective media and confirmed by qPCR targeting the *uidA* gene. Forty isolates were confirmed, of which ten (25%) carried at least one resistance gene as determined by a panel of 57 primer sets. All ten isolates carrying antimicrobial resistance-associated genes (100%) harbored determinants associated with multidrug resistance. The most frequently detected genes were *blaTEM* (80%), *aadA17* (90%), and *intI1-a-marko* (80%). A high prevalence of *qacEΔ1* (80%) was also observed. At least one class 1 integron-associated gene was detected in all resistant isolates and those carrying both integron variants harbored the highest numbers of resistance genes (14–19). Resistance gene profiles in pigeon isolates mirrored those from poultry, suggesting wild birds as potential vectors. The correlation between detected resistance genes and farm antimicrobial use (enrofloxacin, trimethoprim/sulfonamide combinations) indicates strong selective pressure. These findings suggest that poultry farms may serve as environments supporting the persistence and dissemination of antimicrobial resistance-associated genes in *E. coli* and underscore the need for enhanced biosecurity and antimicrobial stewardship within a One Health framework.

## Simple Summary

Antimicrobial resistance (AMR) is one of the most serious global health threats today. The overuse of antibiotics in poultry farming can lead to the emergence of bacteria that no longer respond to treatment. This study investigated the presence of antibiotic-resistant *Escherichia coli* on a commercial chicken farm (egg production) in the West Kazakhstan region. A total of 100 samples were collected from chickens (cloacal swabs, fresh feces, and internal organs), the farm environment, and wild pigeons. The samples were analyzed using cultural and genetic methods to detect resistance genes. Of the 40 *E. coli* isolates confirmed, ten (25%) carried at least one resistance gene, and all ten carried resistance genes associated with resistance to multiple antimicrobial classes. The most common resistance genes were *blaTEM* (80%), *aadA17* (90%), and *int1-a-marko* (80%). Genes providing resistance to disinfectants (*qacEΔ1*) were also found in 80% of the resistant isolates. Importantly, similar resistance genes were detected in *E. coli* from pigeons, suggesting that wild birds may act as carriers and contribute to the spread of resistant bacteria between farms and the environment. These findings highlight the need for responsible antibiotic use and improved biosecurity measures on poultry farms to protect both animal and human health.

## 1. Introduction

Antimicrobial resistance (AMR) is recognized as one of the most critical global health threats of the 21st century. The World Health Organization (WHO) has declared AMR a top priority, warning that without effective intervention, common infectious diseases may once again become untreatable, leading to increased morbidity, mortality, and healthcare costs worldwide [1]. The emergence and rapid spread of resistant bacteria, particularly among the family Enterobacteriaceae, have been driven largely by the misuse and overuse of antimicrobial agents in human medicine, veterinary practice, and food production livestock [2].

Poultry production is one of the fastest-growing livestock sectors globally and represents a major consumer of veterinary antimicrobials [3]. In many countries, antibiotics are administered not only for therapeutic purposes but also for routine prophylaxis and growth promotion, creating strong selective pressure for the emergence and persistence of resistant bacterial strains. *Escherichia coli*, a commensal bacterium of the intestine of humans and animals, is widely used as an indicator organism for monitoring AMR due to its ability to acquire and disseminate resistance genes through horizontal gene transfer [4].

The problem of antimicrobial resistance in poultry production is particularly acute in low- and middle-income countries, where regulatory frameworks for antimicrobial use may be less stringent and surveillance systems remain underdeveloped [2]. In Kazakhstan, as in many Central Asian countries, the poultry industry has expanded rapidly in recent years to meet domestic demand for meat and eggs. However, comprehensive data on antimicrobial use in livestock production and the molecular epidemiology of AMR in poultry-associated bacteria remain extremely limited. This knowledge gap is significant, as the transmission pathways of resistant food-borne pathogens have been extensively reviewed [5], and similar resistance patterns have been documented in poultry and cattle in Bangladesh [6]. Yet, there is a notable lack of molecular characterization of resistance genes and mobile genetic elements in *E. coli* isolates from poultry farms in the West Kazakhstan region.

To our knowledge, this is the first study in the West Kazakhstan region to simultaneously characterize antimicrobial resistance genes, mobile genetic elements, and disinfectant-associated resistance determinants in poultry-associated *E. coli* isolates from poultry, farm-associated environments, and wild birds within a One Health framework.

Regional surveillance data indicate increasing circulation of extended-spectrum β-lactamase (ESBL)-producing *E. coli* carrying *blaCTX-M*, *blaSHV*, and *blaTEM* genes in clinical settings in Kazakhstan [7]. However, the contribution of poultry production systems to the dissemination of these resistance determinants in Kazakhstan remains insufficiently studied. Furthermore, wild birds, particularly pigeons, have been increasingly recognized as potential reservoirs and vectors of antimicrobial-resistant bacteria, facilitating the spread of resistance genes across different environments and between livestock operations [8]. A recent systematic review revealed that urban-adapted birds, especially pigeons and gulls, frequently carry clinically relevant antimicrobial-resistant bacteria including ESBL-producing *E. coli* [9]. However, studies investigating the genetic relatedness of *E. coli* isolates from poultry and wild birds in Central Asia are scarce.

The characterization of resistance genes and mobile genetic elements is essential for understanding the mechanisms underlying AMR and its potential for horizontal gene transfer. Class 1 integrons (*intI1*) and insertion sequences such as IS26 and IS6100 are known to facilitate the capture, expression, and dissemination of resistance gene cassettes [10,11]. The presence of plasmid-mediated quinolone resistance (PMQR) genes, including *qnrB* and *qepA*, is particularly concerning due to their potential for rapid spread and association with critically important antimicrobials for human medicine [12,13]. High-throughput real-time PCR arrays, such as the panel of 57 primer sets developed by Stedtfeld et al. [14], offer a powerful tool for the simultaneous detection of multiple resistance genes and mobile genetic elements, enabling comprehensive molecular profiling of resistant bacterial isolates.

The aim of this study was to conduct molecular surveillance of antimicrobial resistance-associated genes and mobile genetic elements in *E. coli* isolates obtained from poultry, environmental, feed, water, and wild bird samples collected at a commercial layer poultry farm in the West Kazakhstan region. Wild bird samples consisted of cloacal swabs collected from pigeons located near the poultry feed storage area.

As part of a broader antimicrobial resistance monitoring project conducted in collaboration with the poultry farm, this study was designed as an initial molecular screening investigation to assess the potential circulation of antimicrobial resistance determinants within the farm environment and associated biological sources. The findings were intended to provide baseline data for preliminary risk assessment and future surveillance activities within a One Health framework.

## 2. Materials and Methods

### 2.1. Ethical Statement

The present study was conducted at the Veterinary and Biological Safety Laboratory of Zhangir Khan West Kazakhstan Agrarian Technical University, Kazakhstan.

The study protocols and procedures involving animals were reviewed and approved by the Local Commission on Biological Ethics of Kazakh Scientific Research Veterinary Institute (West Kazakhstan Research Veterinary Station branch). The approval was granted at the meeting held on 22 October 2024 (Protocol No. 1).

All procedures involving experimental animals complied with international ethical standards, including the European Convention for the Protection of Vertebrate Animals Used for Experimental and Other Scientific Purposes and the WHO Guidelines for Ethics Committees Reviewing Biomedical Research, as well as European Commission Recommendation 2007/526/EC on the housing and care of animals used for scientific purposes.

### 2.2. Study Design and Sampling

This cross-sectional study was conducted in the West Kazakhstan region, Republic of Kazakhstan, at a commercial layer poultry farm located in the vicinity of Uralsk city (51.23° N, 51.37° E). Due to confidentiality agreements with the commercial poultry producer, the exact identity and precise location of the farm are not disclosed.

**Figure 1:**
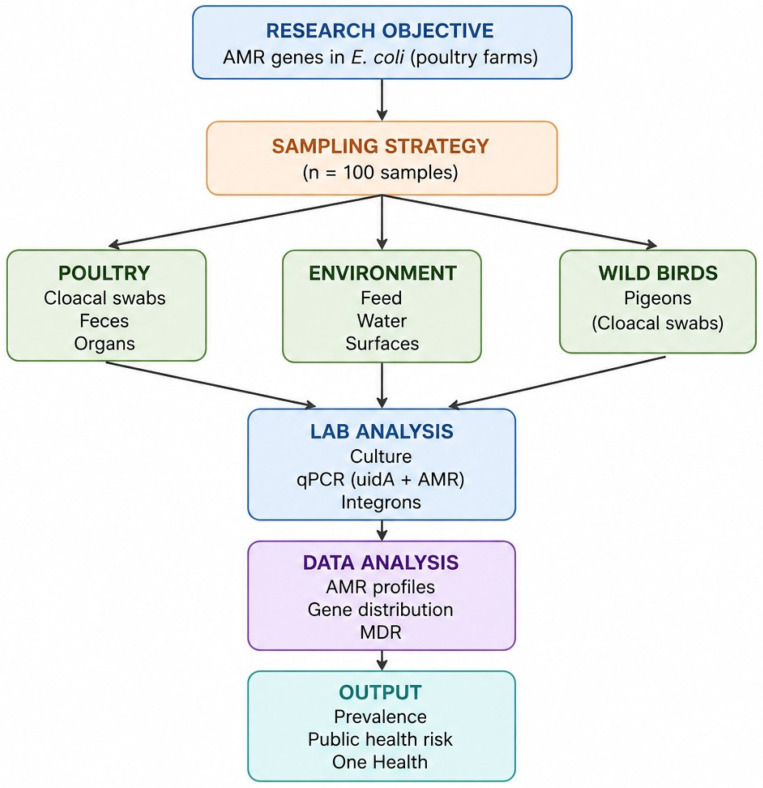
Schematic overview of the study design, sampling strategy, laboratory workflow, and molecular characterization of antimicrobial resistance genes in *Escherichia coli* isolates from poultry, environmental, and wild bird samples.

A schematic overview of the study design and sampling framework is presented in Figure 1.

For confidentiality reasons and in accordance with farm management agreements, the name of the participating farm is anonymized and referred to as a commercial layer poultry farm throughout the manuscript. The farm operated a commercial layer production system and housed Dekalb White laying hens. Adult birds were maintained in conventional four-tier cage batteries equipped with Big Dutchman housing systems under standard commercial management conditions. Each poultry house accommodated approximately 6000–7000 laying hens, while replacement pullets were housed separately. Feed was provided through an automated feeding system using commercially formulated layer rations supplemented with vitamin–mineral premixes, and water was supplied via nipple drinking systems with ad libitum access. Poultry houses were equipped with regulated ventilation, lighting, and sanitation systems in accordance with routine commercial biosecurity practices.

Field sampling was conducted during two visits to capture potential seasonal variation: September 2025 and January 2026. Approximately 50 samples were collected during each sampling visit.

A total of 100 microbiological specimens were collected from the poultry farm, including specimens from internal organs, the environment, feed, water, and wild birds. Each collected specimen (cloacal swab, organ sample, fecal sample, feed, water, or environmental swab) was considered an independent microbiological sample for bacteriological analysis. To obtain a comprehensive representation of the farm environment, specimens were collected from different biological and environmental matrices.

From live birds: Cloacal swab samples were collected from 40 clinically healthy laying hens randomly selected from different cage sections of the poultry house. Sterile cotton swabs moistened with sterile phosphate-buffered saline were gently inserted into the cloaca and rotated before placement into sterile transport tubes. In addition, eight freshly voided fecal samples were collected aseptically from poultry house floor surfaces immediately after defecation to minimize environmental contamination.

From internal organs: Ten birds showing mild clinical signs, including lethargy and ruffled feathers, were provided by farm staff for diagnostic examination. These birds were humanely euthanized according to standard veterinary practice for diagnostic purposes [15]. Post-mortem examinations were performed under aseptic conditions, and samples from the cecum, small intestine, large intestine, and liver were collected for bacteriological analysis.

From the environment: Two environmental samples were collected using sterile swabs moistened with sterile buffer solution from poultry cage floor surfaces and other areas in direct contact with birds, including feeders, drinkers, cages, walls, and floors of poultry houses.

Production inputs: Two drinking water samples were collected from the farm water supply system, and two feed samples containing commercial premix supplements were collected from feeders under aseptic conditions.

Wildlife sampling was conducted to assess the potential role of wild birds in bacterial dissemination within the farm environment. Five clinically normal pigeons (*Columba livia*) observed near the feed storage and processing areas were captured using a net-based trapping method [16]. A total of 10 samples, including cloacal swabs and intestinal samples, were aseptically collected from these birds using the same procedures as for poultry sampling. Most birds were released immediately after sampling to minimize stress, while one pigeon was humanely euthanized for collection of internal organ samples. The limited number of wild birds included in the study reflects the exploratory nature of this preliminary surveillance investigation and the restricted availability of birds for safe capture during the sampling period. Wild bird sampling was conducted during the same farm visits as poultry sampling to ensure temporal comparability between sample sources.

All bird sampling procedures were performed in accordance with standard veterinary ethical practices and with the permission of farm management. Immediately after collection, all samples were placed in sterile labeled containers, transported to the laboratory under refrigerated conditions (2–8 °C), and processed within 24 h to ensure bacterial viability.

### 2.3. Study Farm and Housing Conditions

The study was conducted at a commercial layer poultry farm located in the West Kazakhstan region, Republic of Kazakhstan. The farm housed Dekalb White laying hens maintained in a conventional cage production system equipped with Big Dutchman housing systems (Big Dutchman International GmbH, Vechta-Calveslage, Germany). Adult laying hens were housed in four-tier cage batteries under standard commercial management conditions. The farm included separate poultry houses for replacement pullets and adult layers, with approximately 6000–7000 hens per house.

Feed was produced on-site and provided through automated feeding systems using commercially formulated layer rations supplemented with vitamin–mineral premixes. Water was supplied through automated nipple drinking systems with ad libitum access. Poultry houses were maintained under controlled environmental conditions, including regulated ventilation, temperature, humidity, and lighting. Routine biosecurity measures included sanitation barriers, controlled farm access, and vaccination programs against major poultry infectious diseases.

### 2.4. Microbiological Isolation and Identification

Samples were pre-enriched in 10 mL of Buffered Peptone Water (BPW; Sigma-Aldrich, St. Louis, MO, USA) and incubated at 37 °C for 18–24 h [17]. For internal organ samples, aseptic tissue fragments from the liver, cecum, intestine, and other parenchymal organs were collected using sterile instruments and suspended in sterile physiological saline prior to pre-enrichment. All samples, including internal organs, cloacal swabs, feces, environmental, feed, and water samples, subsequently underwent the same pre-enrichment and culture procedure. For isolation of *E. coli*, a loopful of the pre-enrichment culture was streaked onto MacConkey Agar (MCA; HiMedia, Mumbai, India) and Eosin Methylene Blue Agar (EMB; HiMedia, Mumbai, India) [18]. Plates were incubated at 37 °C for 24–48 h.

Presumptive *E. coli* colonies (lactose-positive on MCA and exhibiting a metallic sheen on EMB) were subcultured onto Tryptic Soy Agar (TSA; HiMedia) to obtain pure cultures [19].

Pure isolates were identified using conventional biochemical tests, including Triple Sugar Iron Agar (TSI), Simmons Citrate, Methyl Red, Voges–Proskauer, indole production, urease, and lysine decarboxylase tests [20].

Isolates with biochemical profiles consistent with *E. coli* (Indole^+^, MR^+^, VP^−^, Citrate^−^, TSI A/A, H_2_S^−^) were stored at −80 °C for further analysis [21].

### 2.5. Molecular Confirmation by qPCR

Molecular confirmation of presumptive isolates was performed by real-time polymerase chain reaction (qPCR) targeting the uidA gene. DNA was extracted from overnight pure cultures by thermal lysis: bacterial colonies were suspended in 100 µL of nuclease-free water, heated at 95 °C for 10 min, and centrifuged, after which the supernatant was used as a template [22,23].

Real-time PCR was performed using a QuantStudio™ 5 Real-Time PCR System (Applied Biosystems, Thermo Fisher Scientific, Waltham, MA, USA). Amplification reactions were carried out in a final volume of 20 µL using BioMaster UDG HS-qPCR Lo-ROX SYBR Green (2×) (Biolabmix, Novosibirsk, Russia), according to the manufacturer’s instruction. Each reaction mixture contained 10 µL of 2× SYBR MasterMix, 0.5 µM of each forward and reverse primer, 2 µL of template DNA, and nuclease-free water to the final volume.

Species-specific primers targeting the uidA gene of *E. coli* were used: uidA-F: 5′-AAA ACG GCA AGA AAA AGC AG-3′ and uidA-R: 5′-ACG CGT GGT TGA GTA GCT GA-3′ (amplicon size: 147 bp) [24]. Thermal cycling conditions consisted of initial denaturation at 95 °C for 5 min, followed by 40 cycles of 95 °C for 15 s and 60 °C for 30 s with fluorescence acquisition. A melting curve analysis (65–95 °C) was performed at the end of amplification to confirm product specificity. Positive controls (*E. coli* ATCC 25922), negative controls, and no-template controls were included in each run. All qPCR reactions were performed in duplicate.

### 2.6. Molecular Screening of Antimicrobial Resistance Genes

The presence of antimicrobial resistance genes was assessed using real-time PCR targeting genes associated with resistance to β-lactams, aminoglycosides, tetracyclines, sulfonamides, fluoroquinolones, macrolides, phenicols, and disinfectants. Mobile genetic elements, including class 1 integrons and insertion sequences, were also investigated.

A panel of 57 primer pairs was used for the detection of antimicrobial resistance genes. Custom standard DNA oligonucleotides (25 nmol scale; catalog no. A15612) were synthesized by Thermo Fisher Scientific (Applied Biosystems, Thermo Fisher Scientific, Waltham, MA, USA) and supplied through ZALMA Ltd. (Almaty, Kazakhstan).

The following genes were included in the analysis: β-lactam resistance genes (*blaTEM*, *blaCTX-M*, *blaCMY*, *blaSHV*, *blaOXA*, *blaACC-1*, *blaDHA*, *blaFOX*, *blaPER*, *blaIMP*, *blaNDM*, *KPC*); aminoglycoside resistance genes (*aadA*, *aadB*, *aadD*, *aphA1*, *aph(3″)-Ia*, *ArmA*, *aacA43*, *strA*, *strB*); tetracycline resistance genes (*tetA*, *tetB*, *tetM*, *tetO*, *tetX*); sulfonamide and trimethoprim resistance genes (*sul1*, *sul2*, *dfrA variants*); quinolone resistance genes (*qnrB*, *qepA*, *oqxA*); *phenicol resistance genes* (*catA1*, *cmlA1*, *floR*); macrolide-lincosamide resistance genes (*ermB*, *ermF*, *mphA*); disinfectant resistance genes (*qac*); and mobile genetic elements, including class 1 integrons (*intI1*), insertion sequences (*IS26*, *IS6100*, *IS6/257*), and plasmid-associated genes (*tra*, *mob*).

Primer sequences (Table S1) were obtained from Stedtfeld et al. (2018) [14]. Real-time PCR was performed in a total reaction volume of 25 µL containing 12.5 µL of BioMaster UDG HS-qPCR (2×) Lo-ROX SYBR Master Mix, primer mix, 5.0 µL of DNA template (3 ng), and nuclease-free water.

Amplification conditions consisted of an initial denaturation at 95 °C for 3 min, followed by 30 cycles of denaturation at 95 °C for 15 s, annealing at 62 °C for 20 s, and elongation at 72 °C for 10 s. Melt curve analysis was performed from 65 °C to 95 °C with 0.5 °C increments to confirm amplification specificity. A threshold cycle (Ct) value of 28 was used as the cutoff for positive amplification, as recommended by the original authors.

All reactions were performed in triplicate. Each run included previously characterized positive control isolates carrying representative antimicrobial resistance genes, as well as negative and no-template controls.

### 2.7. Farm Hygiene and Biosecurity Assessment

A standardized on-site evaluation of husbandry practices and biosecurity measures was conducted at the poultry farm (layer operation) using an observational checklist adapted from FAO biosecurity guidelines [25]. The assessment covered infrastructure, access control, sanitation protocols, health management, and feed/water systems.

#### 2.7.1. Zoohygienic Conditions

Microclimate parameters (temperature, humidity, air velocity, gas composition, dust concentration, illumination) were measured using standard equipment: an August psychrometer (SATO Keiryoki Mfg. Co., Ltd., Tokyo, Japan), a fur aspirator “AM-5M” (MS GO Ekran LLC, Moscow, Russia) with indicator tubes, a thermo-anemometer (AZ-8906, AZ Instrument Corp., Taichung, Taiwan), a handheld luxmeter (model 8581, AZ Instrument Corp., Taichung City, Taiwan), and the gravimetric method with an aspirator (PU-3E, NIKI MLT LLC, Saint Petersburg, Russia), according to previously described methods.

Microclimate parameters. Measurements of microclimate parameters in poultry houses showed values within zootechnical standards: air temperature ranged from 18–20 °C for adult laying hens; relative humidity was maintained at 60–70%; air velocity ranged from 0.2–0.3 m/s in winter and up to 0.5 m/s in summer. Carbon dioxide concentration did not exceed 0.25%, ammonia levels ranged from 5–20 mg/m^3^, hydrogen sulfide concentration was up to 5 mg/m^3^, and dust concentration did not exceed 3.0 mg/m^3^. Illumination was maintained at 10–15 lux during rearing and 15–20 lux for laying hens, with a regulated light regime of 16–17 h of light per day.

Biosecurity measures. The farm implemented comprehensive biosecurity protocols including: a single entry point with disinfection barriers (footbath and wheel dip); an “all-in/all-out” system with 14-day sanitary breaks between production cycles; sequential sanitation of poultry houses after each cycle (litter removal, mechanical cleaning, washing, disinfection with 3% hot solution of “Macrodez” or “Miracid”); insect control using “Perteid 25%”; and rodent control using “Shtorm”. These measures ensured maintenance of high sanitary status and prevention of infectious disease transmission.

Vaccination program. Preventive vaccination was administered from day 0 to 21 against Newcastle disease (La Sota strain), infectious bronchitis (H-120 strain), and infectious bursal disease (“Hipragumboro-GM97”).

#### 2.7.2. Veterinary, Sanitary Measures and Antimicrobial Use (AMU)

Data on antimicrobial use over a 5-year period (2020–2025) were collected from farm records. The assessment also included documentation of vaccination protocols, disinfection procedures, pest control measures, and biosecurity practices, following the methodology recommended by the World Organisation for Animal Health (WOAH) [2] and the European Food Safety Authority (EFSA) [26]. Analysis of farm records over a 5-year period (2020–2025) documented the routine prophylactic use of antimicrobial agents in drinking water. The following antibiotics were used: enrofloxacin (“Enroxil”), trimethoprim/sulfonamide combinations (“Tromexin”), and trimethoprim. For layer operations, antimicrobial prophylaxis was administered during the first 100 days of rearing at a dosage of 10 mg active substance per kg of body weight (equivalent to 0.5–1 mL of 10% solution per 1 L of water).

### 2.8. Statistical Analysis

Descriptive statistics were used to summarize the distribution of *E. coli* isolates and antimicrobial resistance-associated genes among different sample sources. Isolation rates were calculated as percentages based on the number of positive isolates relative to the total number of samples collected from each source. Data visualization and descriptive analyses were performed using Microsoft Excel 2019 (Microsoft Corp., Redmond, WA, USA). Due to the limited number of resistant isolates included in the qPCR screening analysis, no inferential statistical tests were performed.

## 3. Results

### 3.1. Isolation and Identification of E. coli

A total of 100 microbiological specimens were collected from various sources, including internal organs (cecum, *n* = 26; liver, *n* = 10), cloacal swabs (*n* = 40), fresh feces (*n* = 8), environmental samples (*n* = 2), water samples (*n* = 2), feed samples (*n* = 2), and wild bird microbiological specimens obtained from five pigeons (Columba livia, *n* = 10 specimens). From these, 40 *E. coli* isolates were confirmed by qPCR targeting the uidA gene. The distribution of *E. coli* isolates by sample source is presented in Table 1.

Environmental samples included poultry cage floor swabs, water samples from drinking systems, and feed samples mixed with premixes. The highest isolation rate was observed in cloacal swabs (65.0%, 26/40), followed by environmental samples, water, and feed samples (50.0% each), wild birds (40.0%, 4/10), cecum samples (19.2%, 5/26), fresh feces (12.5%, 1/8), and liver samples (10.0%, 1/10).

All 40 isolates were confirmed as *E. coli* by qPCR targeting the *uidA* gene. The specificity of amplification was confirmed by melt curve analysis, which showed a single peak at 80.5 ± 0.3 °C for all positive samples, indicating specific product formation without primer–dimer artifacts. The positive control (*E. coli* ATCC 25922) exhibited consistent amplification, while no amplification was observed in negative controls (no template).

### 3.2. Detection of Antimicrobial Resistance Genes

A panel of 57 primer sets targeting antimicrobial resistance genes and mobile genetic elements was used to screen 40 *E. coli* isolates confirmed by qPCR. Among these, ten isolates (25%) were positive for at least one resistance gene. The resistant isolates were designated as isolates 535, 543v, 544E, and 492, 575, 589, 610, 616, 625, 640.

Representative resistance determinants are summarized in Table 2, while complete qPCR profiles are presented in Supplementary Table S2.

The most frequently detected resistance genes were *aadA17* (90%, 9/10), followed by *blaTEM*, *int1-a-marko*, *qacEΔ1*, *IS26*, *blaCMY*, and *sul2* (80% each). Genes *qnrB46*,*47*,*48* were detected in 70% of isolates, whereas *sul1* and *intI1F165_clinical* were identified in 60% of isolates (Table 3).

A descriptive correspondence was observed between the presence of class 1 integrons (int1-a-marko, *intI1F165_clinical*) and multiple resistance genes. All ten isolates carried at least one integron gene (complete integron profiles are presented in Supplementary Table S2), and isolates with both integron variants (535, 543v, 544E, 575, 616, 640) harbored the highest numbers of resistance genes (14–19 genes). All ten isolates carried resistance genes associated with resistance to three or more antimicrobial classes, indicating the presence of resistance determinants associated with multiple antimicrobial classes.

### 3.3. Association Between Detected AMR Genes and Antimicrobial Use Practices

The resistance genes identified in *E. coli* isolates directly corresponded to the antimicrobials used on the farm (Table 4):

The high prevalence of fluoroquinolone resistance genes (*qnrB46*,*47*,*48* in 70% of resistant isolates) is consistent with the routine use of enrofloxacin on the farm. Similarly, the frequent detection of sulfonamide (*sul1*, *sul2*) and trimethoprim (*dfrA1*, *dfra14*) resistance genes corresponds to the prophylactic administration of trimethoprim/sulfonamide combinations. The presence of β-lactamase genes (*blaTEM*, *blaCMY*, *blaCTX-M*) despite the absence of prophylactic β-lactam use may reflect co-selection with other resistance determinants on mobile genetic elements or environmental contamination.

## 4. Discussion

The present study provides a comprehensive analysis of antimicrobial resistance genes in isolates from a commercial layer poultry farm in Western Kazakhstan. A total of 40 *E. coli* isolates were confirmed by qPCR, of which 10 (25%) harbored at least one resistance gene, and all positive isolates carried resistance gene combinations associated with multidrug resistance.

The highest isolation rate of *E. coli* was observed in cloacal swabs (65.0%), followed by wild birds (40.0%) and fresh feces (12.5%). The detection of *E. coli* in pigeons captured near feed storage areas (40.0%) is particularly noteworthy, as it suggests that wild birds may contribute to bacterial circulation within the farm environment. Similar findings have been reported in other studies, where wild birds were identified as reservoirs and disseminators of antimicrobial-resistant *E. coli* in agricultural settings [27,28,29,30].

This prevalence is comparable to findings from other poultry studies in Central Asia and Eastern Europe, where resistance rates in *E. coli* range from 20% to 35% [31]. The most frequently detected resistance genes were *blaTEM* (80%), *aadA17* (90%), and *int1-a-marko* (80%). The high prevalence of *blaTEM*, a common broad-spectrum β-lactamase gene, is consistent with global trends indicating its widespread distribution among Enterobacteriaceae in livestock production [32]. The detection of *blaCTX-M* (30%) and *blaCMY* (70%) in this study is of particular concern, as these genes confer resistance to extended-spectrum cephalosporins, which are critically important for human medicine [33]. The presence of ESBL-producing *E. coli* in poultry has been increasingly reported worldwide and represents a potential food safety concern due to the risk of foodborne transmission [34].

Regional surveillance data from the West Kazakhstan region support these findings. According to regional antimicrobial resistance monitoring data obtained within the framework of the present research project and based on anonymized reports from a local sanitary-epidemiological laboratory in Uralsk, clinical isolates collected in 2025 demonstrated increasing circulation of ESBL-producing *E. coli* carrying *blaCTX-M*, *blaSHV*, and *blaTEM* genes in clinical settings. The detection of *blaCTX-M* (30%), *blaTEM* (80%), and *blaSHV-11* (20%) in our poultry isolates reflects this regional trend. In addition, acute intestinal infections among children accounted for 76.2% of more than 5000 registered cases during the first five months of 2025, highlighting the public health relevance of antimicrobial resistance in enteric bacteria.

The high prevalence of *aadA17* (90%) among resistant isolates indicates widespread aminoglycoside resistance, likely driven by co-selection with other resistance genes located on the same mobile genetic elements, as aminoglycosides are not commonly used in poultry [35,36]. Tetracycline resistance genes (*tetA*, *tetB*, *tetO*) were detected at moderate frequencies (30% each). Tetracyclines have been widely used in livestock for decades, and the continued detection of tetracycline resistance genes reflects their long-term persistence in the farm environment, even after reduced usage [37].

Fluoroquinolone resistance genes (*qnrB46*,*47*,*48*, *qepA_1_2*) were detected in 70% and 30% of resistant isolates, respectively. The presence of plasmid-mediated quinolone resistance (PMQR) genes is particularly concerning because they can facilitate the spread of reduced susceptibility to fluoroquinolones even in the absence of direct selective pressure [13]. The routine prophylactic use of enrofloxacin on the farm during the first 100 days of rearing may contribute to the maintenance of these PMQR genes within the farm environment [38].

Sulfonamide resistance genes (*sul1*, *sul2*) were detected in 70% of resistant isolates, and trimethoprim resistance genes (*dfrA1*, *dfra14*) were detected in 60% and 40%, respectively. These findings are consistent with the documented use of trimethoprim/sulfonamide combinations (“Tromexin”) on the farm. The high prevalence of these genes is consistent with other studies reporting widespread sulfonamide and trimethoprim resistance in poultry-associated *E. coli* [39].

The detection of *qacEΔ1* in 80% of resistant isolates is of particular interest. The farm routinely uses disinfectants containing quaternary ammonium compounds (QACs), such as “Macrodez” and “Miracid”. The presence of *qacEΔ1* has been associated with reduced susceptibility to QACs and may contribute to the survival and persistence of resistant bacteria in disinfected farm environments. This finding highlights the potential role of biocide use in co-selecting for antibiotic resistance [40].

All ten resistant isolates carried at least one class 1 integron gene (*int1-a-marko* or *intI1F165_clinical*), and isolates with both integron variants (535, 543v, 544E, 575, 616, 640) harbored the highest numbers of resistance genes (14–19 genes). Class 1 integrons are well-established hotspots for the capture and expression of resistance gene cassettes and play a central role in the dissemination of multidrug resistance among Gram-negative bacteria [37]. The presence of insertion sequences (*IS26*, *IS6100*) further supports the potential for horizontal gene transfer [41].

A descriptive correspondence was observed between the antimicrobials used on the farm and the corresponding resistance genes detected in *E. coli* isolates. Enrofloxacin use was associated with the presence of qnrB46,47,48 and qepA_1_2; trimethoprim/sulfonamide use correlated with *dfrA1*, *dfra14*, *sul1*, and *sul2;* and *β-lactamase genes* (*blaTEM*, *blaCMY*, *blaCTX-M*) were detected despite the absence of prophylactic β-lactam use, suggesting co-selection with other resistance determinants or environmental contamination [36]. These findings align with the growing body of evidence demonstrating that antimicrobial use in food-producing animals selects for resistance in commensal and pathogenic bacteria, with potential implications for human health [42].

The detection of similar resistance gene profiles in *E. coli* isolates from poultry and pigeons (e.g., *blaTEM*, *int1-a-marko*, *aadA17*, *qacEΔ1*) suggests that wild birds may act as environmental carriers and contribute to the circulation of antimicrobial resistance genes within and between poultry farms [42]. This finding underscores the need for enhanced biosecurity measures to limit wild bird access to feed storage areas and poultry houses [25].

The detection of *E. coli* in feed samples may reflect environmental or fecal contamination occurring during feed storage, handling, or exposure to wild birds and farm-associated dust particles.

The concordance between resistance gene profiles in poultry isolates and clinical isolates from the same region supports the One Health concept, recognizing the interconnectedness of human, animal, and environmental health. The high proportion of acute intestinal infections in children, coupled with the detection of ESBL-associated resistance genes in poultry-associated *E. coli*, suggests that poultry products may contribute to the dissemination of resistant bacteria through the food chain [43]. This study contributes to the national antimicrobial resistance surveillance efforts under the “Roadmap for Containment of Antimicrobial Resistance (2023–2027)” and aligns with European Food Safety Authority (EFSA) and National Antimicrobial Resistance Monitoring System (NARMS) data, which report high levels of fluoroquinolone and tetracycline resistance in poultry-associated *E. coli* across multiple countries [44].

Several limitations should be acknowledged. First, this study was conducted at a single poultry farm, which limits the generalizability of the findings to other farms or regions. Second, phenotypic antimicrobial susceptibility testing was not performed; therefore, the detected resistance genes indicate the genetic potential for antimicrobial resistance rather than confirmed phenotypic resistance. Finally, the absence of direct comparison with human clinical isolates limits assessment of potential zoonotic transmission pathways. Environmental, feed, and water sampling was exploratory in nature and intended to provide preliminary screening data rather than statistically representative prevalence estimates.

## 5. Conclusions

This study demonstrated the presence of *Escherichia coli* carrying multiple antimicrobial resistance determinants and mobile genetic elements in a commercial poultry farm in Western Kazakhstan. All resistant isolates carried resistance determinants associated with multidrug resistance and frequently carried integron-associated resistance determinants, suggesting a high potential for horizontal dissemination within poultry production environments.

The co-occurrence of resistance genes, class 1 integrons, and insertion sequences suggests substantial potential for horizontal gene transfer and persistence under antimicrobial and disinfectant selective pressure. These findings provide important baseline molecular surveillance data for Kazakhstan and support the implementation of integrated One Health-based antimicrobial stewardship and biosecurity strategies under commercial poultry production conditions.

These findings suggest that long-term prophylactic use of critically important antimicrobials in poultry production exerts selective pressure, favoring the persistence and dissemination of corresponding resistance genes in *E. coli* populations.

## Figures and Tables

**Table 1:** Distribution of *E. coli* isolates by sample source.

Source	Detection Site	Number of Microbiological Specimens	Number of Confirmed *E. coli* Isolates	Isolation Rate (%)
Cloacal swabs	Live birds	40	26	65.0
Wild birds (pigeons)	Feed storage area	10	4	40.0
Fresh feces	Poultry house floor	8	1	12.5
Cecum, small intestine, and large intestine	Internal organs	26	5	19.2
Liver	Internal organs	10	1	10.0
Environmental samples	Poultry cage floor surfaces	2	1	50.0
Water	Drinking water system	2	1	50.0
Feed	Feed mixed with premixes	2	1	50.0
Total	—	100	40 isolates	40.0

**Table 2:** Summary of resistance gene profiles in *E. coli* isolates.

№	Isolate	Total Detected Resistance Determinants (Including MGEs)	Representative Resistance Genes and MGEs Detected
1	535	19	*blaTEM*, *blaCMY*, *aadA17*, *sul1*, *qnrB*, *qepA*, *floR*, *mphA*, *qacEΔ1*, *IS26*
2	543v	15	*blaTEM*, *blaCMY*, *tetA*, *aadA17*, *sul1*, *qnrB*, *floR*, *qacEΔ1*, *IS26*
3	544E	16	*blaTEM*, *blaCTX-M*, *tetB*, *qnrB*, *qepA*, *dfrA1*, *qacEΔ1*, *IS6100*
4	492	9	*blaTEM*, *ArmA*, *aadA17*, *tetO*, *sul1*, *IS26*
5	575	15	*blaTEM*, *blaCMY*, *tetA*, *aadA17*, *sul1*, *qnrB*, *floR*, *qacEΔ1*
6	589	15	*blaCMY*, *blaSHV-11*, *aadA17*, *sul1*, *qnrB*, *qepA*, *floR*, *mphA*, *qacEΔ1*, *IS6100*
7	610	6	*blaCMY*, *blaCTX-M*, *tetB*, *qnrB*, *sul2*, *qacEΔ1*
8	616	15	*blaTEM*, *blaCMY*, *tetA*, *aadA17*, *sul2*, *dfrA1*, *qacEΔ1*, *IS6100*
9	625	9	*blaTEM*, *ArmA*, *aadA17*, *tetO*, *sul1*, *IS26*
10	640	16	*blaTEM*, *blaCTX-M*, *aadA17*, *dfrA14*, *qnrB*, *qepA*, *qacEΔ1*, *IS6100*

Note: Only representative resistance genes and mobile genetic elements (MGEs) are shown. Total counts and antimicrobial resistance classes were determined based on the complete qPCR profiles provided in Supplementary Table S2. Letter suffixes in isolate identifiers represent internal laboratory coding and do not indicate different phenotypes.

**Table 3:** Most frequently detected resistance genes in *E. coli* isolates (*n* = 10).

№	Gene	Frequency (*n* = 10)	Resistance Group
1	*aadA17*	9 (90%)	Aminoglycoside
2	*blaTEM*	8 (80%)	β-lactamase
3	*int1-a-marko*	8 (80%)	Class 1 integron
4	*qacEΔ1*	8 (80%)	Disinfectant resistance
5	*IS26*	8 (80%)	Insertion sequence
6	*blaCMY*	8 (80%)	AmpC β-lactamase
7	*sul2*	8 (80%)	Sulfonamide
8	*qnrB46*,*47*,*48*	7 (70%)	Fluoroquinolone
9	*sul1*	6 (60%)	Sulfonamide
10	*intI1F165_clinical*	6 (60%)	Class 1 integron

Note: Only genes detected in ≥60% of isolates are shown.

**Table 4:** Correspondence between AMU and detected resistance genes.

Antimicrobial Class	Antibiotics Used	Detected Resistance Genes	Frequency (%)
Fluoroquinolones	Enrofloxacin	*qnrB46*,*47*,*48*, *qepA_1_2*	70%, 30%
Sulfonamides	Trimethoprim/sulfonamide	*sul1*,*sul2*	70%, 70%
Trimethoprim	Trimethoprim, Tromexin	*dfrA1*, *dfra14*	60%, 40%
β-lactams	(not used prophylactically)	*blaTEM*, *blaCMY*, *blaCTX-M*	90%, 70%, 30%

## Data Availability

The original data presented in this study are included in the article and Supplementary Materials. Further inquiries can be directed to the corresponding author.

## Supplementary Materials

The following supporting information can be downloaded at: https://www.mdpi.com/article/10.3390/biology15110877/s1, Table S1: Primer sequences for detection of antibiotic resistance genes in *Escherichia coli*; Table S2: Complete antimicrobial resistance gene and mobile genetic element profiles of *Escherichia coli* isolates detected by qPCR array.

## Abbreviations

The following abbreviations are used in this manuscript:

AMR	Antimicrobial resistance
AMU	Antimicrobial use
BPW	Buffered Peptone Water
CI	Confidence interval
CLSI	Clinical and Laboratory Standards Institute
Ct	Cycle threshold
EFSA	European Food Safety Authority
EMB	Eosin Methylene Blue Agar
ESBL	Extended-spectrum β-lactamase
FAO	Food and Agriculture Organization
MCA	MacConkey Agar
MDR	Multidrug resistance
MGE	Mobile genetic elements
MLST	Multilocus sequence typing
MR	Methyl red
NARMS	National Antimicrobial Resistance Monitoring System
PCR	Polymerase chain reaction
PMQR	Plasmid-mediated quinolone resistance
QAC	Quaternary ammonium compound
qPCR	Real-time polymerase chain reaction
TSA	Tryptic Soy Agar
TSI	Triple Sugar Iron Agar
VP	Voges–Proskauer
WGS	Whole-genome sequencing
WHO	World Health Organization
WOAH	World Organisation for Animal Health
