# Supplementary material for: Occurrence and Antimicrobial Resistance Profiles of Escherichia coli Isolated from Commercial Poultry Farm in West Kazakhstan
Source: Biology (Basel). 2026 Jun 2;15(11):877. doi: 10.3390/biology15110877 (PMC13256065; doi:10.3390/biology15110877)
Supplement: Supplementary file 1 [file biology-15-00877-s001.zip › Table S1.pdf]

**Table S1. Primer sequences for detection of antibiotic resistance genes in *Escherichia coli***

*Note:* All primer sequences were obtained from Stedtfeld et al. (2018) [1] (Supplementary Table S4).

**Part 1 (Rows 1–28)**

| No. | Gene | Primer number | Forward primer (5′→3′) | Reverse primer (5′→3′) |
|---|---|---|---|---|
| 1 | aph(3")-ia | 435 | TAACAGCGATCGCGTATTTCG | TCCGACTCGTCCAACATCAATA |
| 2 | tra-A | 1526 | AAGTGTTCAGGGTGCTTCTGCGC | GTCATGTACATGATGACCAAAA |
| 3 | aadA5 | 98 | ATCACGATCTTGCGATTTTGCT | CTGCGGATGGGCCTAGAAG |
| 4 | aadA10 | 426 | ACAGGCACTCAACGTCATCG | CGCGGAGAACTCTGCTTTGA |
| 5 | blaCTX-M | 162 | GCGATAACGTGGCGATGAAT | GTCGAGACGGAACGTTTCGT |
| 6 | ampC/blaDHA | 112 | TGGCCGCAGCAGAAAGA | CCGTTTTATGCACCCAGGAA |
| 7 | blaFOXnew | 1125 | CCTACGGCTATTCGAAGGAAGATAAG | CCGGATTGGCCTGGAAGC |
| 8 | erm(B) | 804 | GAACACTAGGGTTGTTCTTGCA | CTGGAACATCTGTGGTATGGC |

| No. | Gene | Primer number | Forward primer (5′→3′) | Reverse primer (5′→3′) |
|---|---|---|---|---|
| 9 | int1-a-marko | 336 | CGAAGTCGAGGCATTTCTGTC | GCCTTCCAGAAAACCGAGGA |
| 10 | aadA2 | 97 | ACGGCTCCGCAGTGGAT | GGCCACAGTAACCAACAAATCA |
| 11 | aadA16 | 427 | ACGGTGGCCTGAAGCC | GAATTGCAGTTCCCGTCTGG |
| 12 | imp-marko (blaIMP-1) | 324 | GGAATAGAGTGGCTTAATTC | GGTTTAACAAAACAACCACC |
| 13 | oqxA | 1577 | GAGTCAACCTACCTCCACTATCA | GCTGCGAGTTATCCAGCAG |
| 14 | tetA | 180 | CTCACCAGCCTGACCTCGAT | CACGTTGTTATAGAAGCCGCATAG |
| 15 | aphA1 | 170 | TGAACAAGTCTGGAAAGAAATGCA | CCTATTAATTTCCCCTCGTCAAAAA |
| 16 | mobA | 1524 | GCTTCCCGTAACGAGGTAGT | CCTTGAACGGTATCAGCACG |
| 17 | ampC | 1505 | CTGGCGCATACCTGGATTAC | GCCAGTTCAGCATCTCCCA |
| 18 | strB | 177 | GCTCGGTCGTGAGAACAATCT | CAATTTCGGTCGCCTGGTAGT |

| No. | Gene | Primer number | Forward primer (5′→3′) | Reverse primer (5′→3′) |
|---|---|---|---|---|
| 19 | aadA17 | 428 | TGTACGGCTCCGCAGTG | CACGGAATGATGTCGTCGTG |
| 20 | NDM new | 362-n2-25-15 | GGCCACACCAGTGACAATATCA | CAGGCAGCCACCAAAAGC |
| 21 | qnrB46,47,48 | 1579 | CGACGTTCAGTGGTTCAGATCTC | GCCAAGCCGCTCCATGAG |
| 22 | dfra14 | 600 | CGGATCATGTCATTGTTTCAGG | ATGTTAGAGGCGAAGTCTTGG |
| 23 | IS26 | 1546 | ATGGATGAAACCTACGTGAAGGTC | CGGTACTTAATCTGTCGGTGTTCA |
| 24 | mphA | 812 | TCAGCGGGATGATCGACTG | GAGGGCGTAGAGGGCGTA |
| 25 | catA1 | 130 | GGGTGAGTTTCACCAGTTTTGATT | CACCTTGTCGCCTTGCGTATA |
| 26 | blaSHV-11 | 1110 | TTGACCGCTGGGAAACGG | TCCGGTCTTATCGGCGATAAAC |
| 27 | qepA_1_2 | 1201 | GGGCATCGCGCTGTTC | GCGCATCGGTGAAGCC |
| 28 | dfrA1 | 58 | GGAATGGCCCTGATATTCCA | AGTCTTGCGTCCAACCAACAG |

**Part 2 (Rows 29–55)**

| No. | Gene | Primer number | Forward primer (5'→3') | Reverse primer (5'→3') |
|---|---|---|---|---|
| 29 | *erm(F)* | 23 | CAGCTTTGGTTGAACATTTACGAA | AAATTCCTAAAATCACAACCGACAA |
| 30 | IS6/257 | 1560 | ATATCGTGCCATTGATGCAGAG | ACCATTGCTACCTTCGTTGAAG |
| 31 | *ArmA* | 405 | TCTTCGACGAATGAAAGAGTCG | GCTAATGGATTGAAGCCACAACC |
| 32 | *floR* | 913 | AACCCGCCCTCTGGATCA | GCCGTCGAGAAGAAGACGAA |
| 33 | *blaOXA10* | 1506 | CGACCGAGTATGTACCTGCTTC | TCAAGTCCAATACGACGAGCTA |
| 34 | *dfrA12* | 59 | CCTCTACCGAACCGTCACACA | GCGACAGCGTTGAAACAACTAC |
| 35 | *aadE* | 174 | TACCTTATTGCCCTTGGAAGAGTTA | GGAACTATGTCCCTTTTAATTCTACAATCT |
| 36 | *IS6100* | 1561 | CGCACCGGCTTGATCAGTA | CTGCCACGCTCAATACCGA |
| 37 | *tetX* | 196 | AAATTTGTTACCGACACGGAAGTT | CATAGCTGAAAAAATCCAGGACAGTT |
| 38 | *cmlA1* | 127 | TAGGAAGCATCGGAACGTTGAT | CAGACCGAGCACGACTGTTG |

| No. | Gene | Primer number | Forward primer (5'→3') | Reverse primer (5'→3') |
|---|---|---|---|---|
| 39 | *blaTEM* | 1512 | CGCCGCATACACTATTCTCAG | GCTTCATTCAGCTCCGGTTC |
| 40 | *pcoA* | 1303 | TGGCGTATGGAGTTTCAATGC | GAATAATGCCGTGCCAGTGAA |
| 41 | *sul1 NEW* | 363 | GCCGATGAGATCAGACGTATTG | CGCATAGCGCTGGGTTTC |
| 42 | *intI1F165_clinical* | 359 | CGAACGAGTGGCGGAGGGTG | TACCCGAGAGCTTGGCACCCA |
| 43 | *aacA43* | 423 | CTTGGCCTACATTAGATTCAGCTC | GCTCTCAATCTTTGATAGGAGCAG |
| 44 | *strA* | 176 | CCGGTGGCATTTGAGAAAAA | GTGGCTCAACCTGCGAAAAG |
| 45 | blaACC-1 | 28 | CACACAGCTGATGGCTTATCTAAAA | AATAAACGCGATGGGTTCCA |
| 46 | *tetB* | 181 | AGTGCGCTTTGGATGCTGTA | AGCCCCAGTAGCTCCTGTGA |
| 47 | *silE* | 1304 | GGTGGAAAGTCATCAGAGGATGA | CAAAGCCCAGCAAGGATGC |
| 48 | *sul2* | 133 | TCATCTGCCAAACTCGTCGTTA | GTCAAAGAACGCCGCAATGT |
| 49 | *tetO* | 192 | CAACATTAACGGAAAGTTTATTGTATACCA | TTGACGCTCCAAATTCATTGTATC |

| No. | Gene | Primer number | Forward primer (5'→3') | Reverse primer (5'→3') |
|---|---|---|---|---|
| 50 | *blaPER* | 1517 | GCAAATGAAGCGCAGATGC | GACCACAGTACCAGCTGGTA |
| 51 | *aadB* | 429 | CCTGCTTGGTGGGCAGAC | CGGCACGCAAGACCTCAA |
| 52 | *blaCMY* | 108 | AAAGCCTCATGGGTGCATAAA | ATAGCTTTTGTTTGCCAGCATCA |
| 53 | *KPC* | 1523 | GCCGCCAATTTGTTGCTGAA | GCCGGTCGTGTTTCCCTTT |
| 54 | mcr-1 | 704 | CACATCGACGGCGTATTCTG | CAACGAGCATACCGACATCG |
| 55 | *traN* | 1525 | GCTTGGCGGTCAGCAATT | TTAGGAATAACAATCGCTACACCTTTA |
| 56 | *qacdelta_159old* | 159 | CCCCTTCCGCCGTTGT | CGACCAGACTGCATAAGCAACA |
| 57 | *qac_new_356old* | 356 | GGAGATTTAGCTCATGTAGCTGAAGAA | AAGCTGTTTTATCCCCGTAGCTTTA |
